# Audiology's Nomenclature and Taxonomy Under the Law: Implications for Access and Coverage of Audiology Services

**DOI:** 10.1055/s-0046-1825793

**Published:** 2026-10-06

**Authors:** Stephanie Czuhajewski, Deb Abel, Kim Cavitt

**Affiliations:** 1 Academy of Doctors of Audiology, Frankfort, Kentucky, United States; 2 Audigy, Vancouver, Washington, United States; 3 Audiology Resources, Chicago, Illinois, United States

**Keywords:** audiology scope of practice, medicare reimbursement, professional taxonomy, healthcare regulation, access to audiology services, access to hearing and balance healthcare

## Abstract

Access to audiology services is shaped not only by clinical competence but also by the statutory language that defines the profession under state licensure laws and federal Medicare policy. This article examines how audiology's legal nomenclature and taxonomy influence scope of practice, reimbursement, and patient access to hearing and balance care in the United States. State audiology practice acts and federal Medicare statutes are compared with those governing other clinical healthcare professions to identify structural differences in professional classification and covered services. The analysis demonstrates that many state practice acts have not evolved alongside the profession's transition to doctoral-level education, resulting in outdated definitions that fail to recognize contemporary responsibilities related to diagnosis, management, treatment, prescribing authority, and the use of clinical extenders. At the federal level, Medicare continues to classify audiology primarily as a diagnostic supplier service, creating gaps in coverage for treatment services and limiting participation in modern reimbursement models. These statutory and regulatory inconsistencies contribute to fragmented care, unnecessary administrative burdens, reduced access for Medicare beneficiaries, and underutilization of audiologists' clinical expertise. Modernizing professional nomenclature and taxonomy at both the state and federal levels is presented as a foundational step toward improving access, aligning regulation with contemporary practice, and strengthening hearing healthcare delivery.

## Introduction

Access to audiologists and coverage of audiology services rely heavily on how the practice of audiology is defined (scope of practice) under state laws and how audiology services are identified, described, and classified (scope of services covered) under federal Medicare statutes. State laws widely authorize audiologists to deliver treatment services such as aural rehabilitation, vestibular rehabilitation, and cerumen management, all of which are covered under Medicare Part B. However, Medicare payment statutes only define audiology services as “diagnostic other,” creating a treatment vacuum that forces beneficiaries to either pay privately for covered audiology treatment services when they are delivered by an audiologist, or to pay multiple co-pays to visit multiple (non-audiologist) providers.

The language contained in most state statutes and regulations impacting the delivery of audiology services is also out of step with evidence-based practices and the contemporary education and training of audiologists. The adoption of the Doctor of Audiology (AuD) degree as the first professional degree for entry into clinical practice two decades ago prompted widespread updates to state licensing laws to reflect increased educational requirements. Unfortunately, most licensing laws failed to also modernize the definition of audiology to explicitly include the evaluation, diagnosis, management, and treatment of auditory and vestibular conditions, and other procedures which are consistent with audiologists' training.

When it comes to access to safe, effective, efficient, and affordable healthcare, words matter. The nomenclature and taxonomy used to define the practice of audiology and the description of covered audiology services in state and federal policies are markedly different from language commonly used to describe the scope of practice and scope of services for other similarly trained clinical healthcare professions. Outdated definitions and classifications under the law result in unnecessary delays in treatment, higher costs of care for consumers, increased administrative burdens, and inefficient utilization of providers and services.

The Medicare Audiology Access Improvement Act (MAAIA), if enacted, will redefine audiology within the Medicare system, close coverage gaps, and streamline access to audiology services for Medicare beneficiaries. State audiology practice acts must also be updated consistently to align laws, regulations, and coverage policies with current standards of clinical practice to increase access and improve outcomes for patients throughout the United States. Addressing these structural gaps will require action at both the state and federal levels.

This article examines how the nomenclature and taxonomy embedded in state audiology practice acts and federal Medicare statutes create systemic barriers to access, and identifies the policy priorities that follow from that documentation. This analysis is intentionally bounded: it characterizes existing statutory and regulatory language, compares it systematically across professions, and identifies the classification gaps that the evidence supports—but it does not evaluate the legislative feasibility of specific reforms, assess the political conditions for their passage, or prescribe implementation timelines. Those evaluative functions are addressed in the companion analyses of workforce economics in this issue (Audiology at the Intersection of Labor, Revenue, and Taxonomy and Professional Status of Audiology in Healthcare) and the profession–wide roadmap and strategic sequencing analysis that closes this issue (Audiology in 2050).

## The State of Audiology Profession as Defined by Language in State Audiology Practice Statutes

The scope of practice for clinicians is dictated by state occupational licensing laws. Audiologists are licensed in every U.S. state and territory. Statutes governing audiology, known as audiology practice acts, are enacted by state legislatures to provide consumer protection. These statutes define the services, standards, and safeguards for the practice of audiology and establish minimal requirements and qualifications to become licensed as an audiologist. Administrative rules and regulations, implemented through state licensing bodies, operationalize audiology practice statutes and provide detailed processes and procedures for obtaining and maintaining licensure as an audiologist. Audiology practice acts and regulations are commonly combined with state laws that govern licensure and scope of practice for speech–language pathology (SLP) and/or hearing aid specialists (HAS).

The first Doctor of Audiology (AuD) degree program was established in 1994 at Baylor University. 1 Over the decade that followed, the AuD became the recognized standard for entry into clinical practice as an audiologist. Beginning in 2007, state licensing laws were updated across the nation to require a doctoral degree for newly trained audiologists. Audiologists holding master's degrees conferred prior to 2007 were “grandfathered” in and authorized to continue to practice in every state. The result is a profession in which doctoral–level training has been the entry standard for nearly two decades, yet the statutory language defining audiology's clinical authority in most states has not been updated to reflect what that training represents.

### Common Scope of Practice Traits of Clinical Doctoring Professionals, APPs, and Other Allied Health Professionals

The state-defined scope of practice for non-physician clinical doctoring professionals, advanced practice providers (APPs) (also known as non-physician practitioners or NPPs), and other allied health professionals varies widely from state to state. The Center for Medicare and Medicaid Services (CMS) defines APPs to include clinical nurse specialists, nurse practitioners, nurse midwives, nurse anesthetists, anesthesiology assistants, and physician assistants. 2

An examination of state practice acts, illustrated in Table 1 , shows that most non-physician clinical doctoring professionals and APPs are authorized to perform one or more of the following activities under their scope of practice, in most states: Order diagnostic testing and/or imaging, prescribe medication, and supervise assistants. Other allied health professionals including occupational therapists, speech-language pathologists, and orthotists and prosthetists are also authorized to use and supervise assistants in most states.

**Table 1:** Common scope of practice traits in clinical doctoring professions, advanced practice providers, and other allied health professions as dictated by state practice acts

Profession	Education required for new entrants to practice	Number of US states permitting provider to order additional diagnostic testing	Number of US states permitting provider to order imaging	Number of US states permitting provider to dispense and prescribe medication	Number of US states permitting the use of assistants performing clinical services
Audiologist	Doctorate	2	2	0	8 ^a^
Chiropractor	Doctorate	50	50 37	1	50
Dentist	Doctorate	50	50	50	50
Nurse practitioner b	Master's	27 38	27	27	50
Occupational therapist	Master's	0	0	0	50
Optometrist	Doctorate	50	50	50	50
Physical therapist 39	Doctorate 40	0	12 d	0	50
Physician assistant c	Master's	22	22	22	50
Podiatrist	Doctorate	50	50	50	50
Psychologist	Doctorate	7	7	7 41	50
Speech-language pathologist	Master's	0	12 42	0	50

Notes: ^a^ The use of audiology assistants to perform clinical services is explicitly prohibited in 11 states, 8 states explicitly allow it, and most states do not address the use of audiology assistants. Notably, most state hearing aid dispensing laws prohibit audiology assistants from providing any hearing aid-related clinical services.

b Includes nurse practitioners, nurse midwives, nurse anesthetists, and clinical nurse specialists.

c Includes anesthesiology assistants.

d Physical therapists can order radiographic imaging in 12 states. 43 44

Audiologists are the only clinical doctors who are not authorized to perform any of these three activities (i.e., order diagnostic testing and/or imaging, prescribe medication, and supervise assistants) under the majority of state statutes governing the practice of audiology. Audiologists are not authorized to prescribe medications in any state. As of October 2024, the audiology practice act in the State of Maryland was the first to be amended to explicitly give audiologists authority to order diagnostic radiologic imaging and testing – a development that signals the future in modernization of scope of practice legislation. 3

Restrictive audiology and hearing aid dispensing laws impede audiologists' ability to utilize and supervise assistants to perform clinical tasks across the audiology care continuum in most states. A related but distinct restriction is that 11 states outright prohibit the use of audiology assistants to provide clinical services under the supervision of an audiologist. Nearly every state prohibits audiology assistants from providing hearing aid dispensing or fitting services under the supervision of an audiologist. 4 The prohibition on the use of audiology assistants to perform clinical services serves to increase wait times and cost of care for individuals seeking hearing and balance care.

The competitive consequence of this restriction is most clearly illustrated by examining who state law does permit to supervise fitting and dispensing activity. In Texas—whose regulatory structure is representative of the asymmetry present in many states—a physician licensed by the Texas Medical Board, including an otolaryngologist or ENT specialist, is explicitly exempted from hearing instrument fitter and dispenser licensure requirements under Texas Occupations Code Chapter 402, 5 and may supervise the fitting and dispensing of hearing instruments under the physician exemption without holding an audiology license. Under this pathway, an ENT practice may employ hearing instrument fitters or dispensers operating under physician supervision, delivering fitting and dispensing services across the full scope of that licensure category, with no audiologist involved at any point in the care pathway. Simultaneously, under 16 Tex. Admin. Code §111.92, 6 audiology assistants are explicitly prohibited from engaging in the fitting, dispensing, or sale of hearing instruments under the audiology chapter—meaning that the profession most clinically qualified to oversee hearing care has less supervisory authority over hearing care extenders than the physician whose core competency lies elsewhere. This statutory asymmetry is not an anomaly. It is the predictable consequence of audiology's failure to secure assistant utilization rights commensurate with its clinical scope: in the absence of an audiologist-supervised extender pathway capable of delivering fitting and dispensing services, state law creates a structural opening for physician-owned practices to deploy lower-cost personnel in exactly the clinical space audiology cannot efficiently occupy. The result is a substitution pathway that operates entirely within the law, routes patients through hearing care without audiologist involvement, and is reinforced rather than constrained by the scope restrictions that audiology's own practice acts impose.

Nurse practitioners, physician assistants, 7 dentists, 8 optometrists, 9 physical therapists, 10 podiatrists, 11 and psychologists 12 are recognized as having a role in the delivery of primary healthcare in the United States. More than one-quarter of primary care visits for Medicare beneficiaries are visits to nurse practitioners or physician assistants, 13 but other more specialized practitioners are also increasingly playing a role in primary care. For example, the American Optometric Association notes that optometrists play a significant role in overall health and well-being beyond vision care, including detection of systemic disease and management of its ocular manifestations. That audiologists—alone among doctoral-level clinical professions—are not authorized to perform any of these three benchmark activities (i.e., order diagnostic testing and/or imaging, prescribe medication, and supervise assistants) in the majority of states represents not a reflection of clinical scope, but a failure of statutory language to keep pace with the profession's training, competence, and public health role.

### Audiology's Most Consistent Scope of Practice Expansion: Moving from Audiologist to Dispensing Audiologist

Clinical professions typically seek scope of practice expansions that coincide with advances in their training, education, and qualifications, and that allow the performance of procedures that require skills commensurate with professions and professionals who are similarly trained. Audiologists have successfully advocated to codify the inclusion of cerumen management, vestibular rehabilitation, and intraoperative monitoring into practice acts in a patchwork of states, largely standardizing audiologists' scope of practice throughout the United States. However, audiology's single most consistent scope of practice expansion over the past four decades was to shift from audiologist to dispensing audiologist.

Audiology's trajectory diverged from this pattern in a way that has had lasting structural consequences. Audiologists began dispensing hearing aids for profit in the early 1970s, defying American Speech Language Hearing Association (ASHA) prohibitions on these activities, which ASHA deemed “unethical.” However, ASHA changed its position and approved dispensing for profit to avoid potential antitrust liabilities, in response to the U.S. Supreme Court ruling on the *National Society of Professional Engineers (Society)* v. *United States of America* , 435 U.S. 679 (1978), which found that the Society's ethical code, prohibiting members from submitting competitive bids, violated the Sherman Act. 14 15

This ruling initiated audiology's legislative effort across states to incorporate hearing aid sales, dispensing, and fitting into practice statutes and regulations across the nation, and to abolish requirements that audiologists carry a separate hearing aid dispensing license to provide such services. Oklahoma was the first state to eliminate such requirements, when, in a 1983 opinion, the Oklahoma Attorney General stated, *“…audiologists licensed pursuant to 59 O.S. 1601 et seq. (1981) are exempt from the rules and regulation of the State Board of Health concerning licensure of hearing aid dealers and fitters. As a result, a licensed audiologist may lawfully sell a hearing aid in the State of Oklahoma without holding a license issued by the State Board of Health pursuant to 63 O.S. 1-1750 (1981).”* 16

In most states, the minimum qualifications for selling, dispensing, and fitting hearing aids under their hearing aid licensure acts include obtaining a high school education, reaching 18 years of age, being free of communicable diseases, and avoiding a felony conviction. As of 2024, six states still require audiologists who dispense hearing aids to hold a separate hearing aid dispensing license. This dual licensure requirement doubles the regulation and licensure costs to practice in those states. 17

From a practical perspective, the surrogates and advocates for the profession of audiology have spent the past 20 years increasing the educational and training requirements necessary to practice audiology, while at the same time failing to see these requirements translate into updated scopes of practice across the nation, reflective of a clinical doctoring profession.

### Audiology Defined as the “Nonmedical Application of Principles, Methods, and Procedures…”

Hearing and balance disorders are medical conditions. 18 19 Hearing loss is the third most common chronic condition for adults over the age of 65 years. Auditory and vestibular conditions are also comorbid with other medical conditions such as heart disease, strokes, kidney disease, diabetes, depression, and cognitive decline. 20

Audiologists are recognized experts in the diagnosis and management of hearing and balance disorders in pediatric and adult populations. 21 22 Older adults with hearing loss are at significantly greater risk for falling than those with normal hearing, and falls are the leading cause of injury and injury death in older adults. 23 24 Children with untreated hearing loss experience language deficits that significantly impede their learning and communication abilities. 25 Early detection and intervention of audiologic conditions are essential for good health at all stages of life.

Despite the important role that audiologists play in diagnosing and treating auditory and vestibular disorders, a small number of state audiology practice statutes use the terms diagnose and treat to describe activities that audiologists perform under their scope of practice. 17 Of the state audiology statutes that include the term diagnosis in the definition of the practice of audiology, more than one-third qualify the term as a “nonmedical” diagnosis. Many other states' statutes use the term nonmedical to define or qualify all the activities that audiologists perform.

For example, the New Jersey Statutes Annotated (N.J.S.A.) states the following:

“Practice of audiology means the nonmedical and nonsurgical application of principles, methods, and procedures of measurement, testing, evaluation, consultation, counseling, instruction, and habilitation or rehabilitation related to hearing, its disorders, and related communication impairments for the purpose of nonmedical diagnosis, prevention, identification, amelioration, or modification of these disorders and conditions in individuals or groups of individuals with speech, language, or hearing disabilities, or to individuals or groups of individuals for whom these conditions must be ruled out.” 26

A review of state practice acts governing other non-physician providers such as chiropractors, clinical social workers, nurse practitioners, occupational therapists, optometrists, physical therapists, podiatrists, and psychologists found that state statutes do not use the term “nonmedical” to qualify the type of diagnosis, evaluation, or treatment (when such terms are used) that those professionals deliver under their state-defined scope of practice.

According to the Merriam-Webster Dictionary, the term nonmedical is synonymous with non-clinical, and is defined as: *“Not involving, relating to, used in, or concerned with medicine, or not relating to treatment of a disease or condition.”*

Nonmedical is often used to define administrative (nonmedical) personnel working in a hospital, to describe the recreational (nonmedical) use of drugs such as marijuana, or to define (nonmedical) services such as meal preparation, house cleaning, and transportation when delivered in a home care setting.

Taber's Medical Dictionary defines diagnosis as: *“Expert identification of a disease or syndrome a person has or is believed to have” and “The use of scientific or clinical methods to establish the cause and nature of a person's illness or injury and the functional impairment it produces. The diagnosis forms the basis for patient care.”* 27

Audiologists are widely recognized as diagnosing and treating providers by government and private payers. In fact, the American Medical Association (AMA) has created Current Procedural Terminology (CPT) codes specifically for documenting audiology diagnostic and treatment services. 28 “Medical” is an intrinsic component of the meaning of diagnosis and treatment. 29 Therefore, the terms “nonmedical diagnosis,” “nonmedical treatment,” and “the nonmedical application of principles, methods, and procedures…” as they are used to describe auditory and vestibular diagnoses and treatments provided by audiologists under their state practice statutes are misnomers that foster public, payer, and interprofessional perceptions of audiology services as merely technical or functional, rather than clinical in nature.

### Summary

State licensure language for audiologists unfairly and inappropriately includes the qualifier “nonmedical.” While the origin of the use of this term to define audiology services is unclear, it has been perpetuated through state legislative processes in which organized medicine has historically advocated for restrictive definitions of audiology's scope.

### The Terms “Prescribe [Hearing Aids]” and “Manage” Are Omitted from Most State Audiology Practice Acts

Most state audiology practice acts do not include the terms “prescribe” or “manage”—omissions with direct consequences for reimbursement authority and clinical recognition. For example, the implementation of the Over-the-Counter (OTC) Hearing Aid Act of 2017 by the U.S. Food and Drug Administration (FDA) in 2022 resulted in the creation of a new prescription category of hearing aids, eliminating federal preemptions regulating the sale of traditional air conduction hearing aids, which had been in place for 50 years, immediately delegating authority to regulate the sale and use of prescription hearing aids to the states.

When the FDA prescription hearing aid regulations went into effect, most state laws did not address the act of “prescribing” hearing aids under audiology's scope of practice or regulation. Prior to that time, traditional hearing aids were classified as restricted medical devices rather than prescription devices, rendering the act of prescribing the hearing aid unnecessary. Since 2022, most state governments have taken action to affirm that audiologists are authorized to “prescribe” and/or “order” prescription hearing aids under their scope of practice through regulations, or official legal determinations by state licensing boards or attorneys general. However, fewer than 15 states explicitly include “prescribe” as part of the definition of audiology in their audiology practice act. 17 This ambiguity could increase legal liability for providers and result in additional authorization requirements or other barriers to reimbursement.

Audiology practice statutes in 44 state jurisdictions exclude the term “manage” from the definition of the practice of audiology—an omission with direct and consequential implications for reimbursement authority. Evaluation and management (E/M) services, billed under CPT codes 99202–99215, are the primary mechanism through which diagnostician-centered healthcare professions—physicians, nurse practitioners, physician assistants, and others—capture the economic value of clinical assessment, diagnostic reasoning, care planning, and patient counseling. These are not procedural codes tied to a specific test or intervention; they are recognition codes that compensate the professional judgment exercised during a clinical encounter. Because audiologists routinely perform each of these functions—determining appropriate diagnostic protocols, interpreting findings, developing management plans, and counseling patients on treatment options—their exclusion from E/M billing means that a substantial portion of audiologic cognitive labor is performed without independent reimbursement recognition. The audiologist's clinical reasoning is compensated indirectly, if at all, through procedure codes that capture the technical component of care while leaving its interpretive and managerial dimensions economically invisible. Where state practice acts do not define audiology to include management of auditory and vestibular conditions, this exclusion is not merely a billing inconvenience, it is a statutory condition that structurally subordinates professional judgment to procedural throughput, with downstream consequences for how audiology is valued, deployed, and sustained as a workforce. The labor economics implications of this classification are examined elsewhere in this issue (see Audiology at the Intersection of Labor, Revenue, and Professional Taxonomy).

Maryland and Arkansas became, respectively, the fifth and sixth states in the nation to include the term “manage” and the ninth and tenth states to include “prescribe” in their audiology practice acts in 2024 and 2025 when their legislatures enacted legislation to modernize the states' audiology practice acts, updating the definition of audiology to include the following activities 30 :

- Evaluate, diagnose, manage, and treat auditory or vestibular conditions of the human ear;
- Prescribe, order, sell, dispense, or fit hearing aids in an individual for the correction or relief of a condition for which hearing aids are worn;
- Prescribe, order, sell, dispense, or externally fit a sound processor to an osseointegrated device for the correction or relief of a condition for which osseointegrated devices are worn;
- Prescribe, order, sell, dispense, or externally fit a sound processor to a cochlear implant for the correction or relief of a condition for which cochlear implants are worn;
- Conducting of health screenings;
- The removal of a foreign body from the external auditory canal that is not impacted to the point where it requires anesthesia;
- The removal of cerumen from the external auditory canal that is not impacted to the point where it requires anesthesia;
- The ordering of cultures and bloodwork testing as it relates to the auditory or vestibular conditions in the human ear;
- The ordering and performing of in-office nonradiographic scanning or imaging of the external auditory canal; and
- The ordering of radiographic imaging as it relates to the auditory or vestibular conditions in the human ear.

The scope elements codified in Maryland and Arkansas closely parallel the clinical competencies required for AuD graduation under both CAA (Council on Academic Accreditation for Audiology and Speech-Language Pathology) and ACAE (Accreditation Commission for Audiology Education) standards. That the majority of states do not yet authorize the activities these standards require graduates to perform represents a systemic misalignment between educational credentialing and regulatory recognition—one examined in detail in the companion analysis of AuD education (Shaping the Future of Audiology) in this issue.

These statutes represent the most comprehensive statutory modernization of audiology scope of practice in recent decades and provide a direct template for other states seeking to align licensure language with the full competency expectations of doctoral-level audiology training.

### Medicare Taxonomy: Scope of Services and Coverage Requirements Impact Beneficiary Access to Audiology Services

Medicare's classification of audiology services has never been updated to reflect the profession's transition to doctoral-level practice. Since the program's inception through the Social Security Amendments of 1965, audiology services have been categorized under Section 1861(s)(3) of the Social Security Act 31 as “other diagnostic tests”—a designation that limits Medicare coverage to diagnostic services and excludes the rehabilitative, therapeutic, and chronic-care services that constitute a substantial portion of contemporary audiologic practice. The 1994 Social Security Act Amendments defined audiology services and introduced the category of “qualified audiologist” for the first time—notably, in the same year the first AuD students entered training at Baylor University. Despite this definitional update, the diagnostic-only classification was preserved, and audiology's statutory position within Medicare has remained structurally unchanged in the three decades since. The consequences of that classification for beneficiary access, professional authority, and workforce sustainability are the focus of this section.

### The 1994 Definition and Its Limits

The 1994 amendments defined audiology services to include the following:

“…hearing and balance assessment services furnished by a qualified audiologist as the audiologist is legally authorized to perform under State law (or the State regulatory mechanism provided by State law), as would otherwise be covered if furnished by a physician.” 31

According to CMS, hearing and balance assessment services also include tests of tinnitus, auditory processing, and surgically implanted devices. Notably, audiology services delivered by audiologists do not require physician supervision as a condition of Medicare reimbursement—a provision that recognizes a degree of independent practice authority for diagnostic services. However, this supervisory exemption does not confer the full provider status or reimbursement authority associated with autonomous clinical professions. As the sections that follow document, audiologists remain classified as suppliers of diagnostic services rather than as limited license practitioners, a distinction that restricts access to the billing mechanisms through which clinical judgment is independently recognized and compensated.

### The Treatment Vacuum

The most consequential feature of Medicare's audiology classification is not what it permits but what it excludes. Coverage of audiology services under Medicare Part B does not extend to treatment services—including vestibular rehabilitation, cerumen management, and aural rehabilitation—despite the fact that these services are covered by Medicare when delivered by other providers, including SLPs, physical therapists, physicians, physician assistants, and nurse practitioners. 32 This asymmetry means that a beneficiary requiring vestibular rehabilitation following a diagnosis of benign paroxysmal positional vertigo may receive covered treatment from a physical therapist but not from the audiologist who diagnosed the condition and is trained to manage it.

Because Medicare coverage statutes authorize audiologists to diagnose auditory and vestibular disorders, yet prohibit audiologists from delivering treatment services to address those conditions, Medicare beneficiaries are faced with an expensive conundrum. They must either pay privately to receive Medicare–covered treatment services (e.g., cerumen removal, aural rehabilitation, and vestibular rehabilitation) from their audiologist or they must make multiple office visits and incur multiple co–payments to access those services through other providers (e.g., a nurse practitioner, speech pathologist, physical therapist). This issue––grounded in Section 1861 of the Social Security Act–––is examined in greater detail elsewhere in this issue (see Audiology at the Intersection of Labor, Revenue, and Taxonomy).

Medicare is additionally statutorily prohibited from covering hearing aids or examinations for the purpose of prescribing, fitting, or changing hearing aids. 33 While this exclusion is distinct from the treatment vacuum described above, its practical effect compounds the coverage gap: beneficiaries navigating hearing loss may find that neither the diagnostic workup leading to amplification nor the rehabilitative services following it are fully covered within a single provider relationship.

### Exclusion from Value-based and Outcome-based Payment Models

Because Medicare coverage of audiology services is restricted to diagnostics, patient outcomes attributable to audiologic care cannot be systematically measured within the program's administrative structures. Audiologists are, therefore, routinely excluded from outcome-based reimbursement incentives and novel Medicare payment models—including value-based care arrangements, alternative payment models, and bundled payment structures—that are increasingly central to how Medicare rewards clinical quality and efficiency across healthcare professions. This exclusion is not merely a current billing inconvenience, it represents a long-term strategic vulnerability. As Medicare continues to shift reimbursement weight toward value-based models, professions whose services are restricted to procedure-based diagnostics will have diminishing access to the payment mechanisms that reward longitudinal care management, patient outcomes, and care coordination. The implications of this structural exclusion for audiology's long-term economic positioning and workforce sustainability are reported elsewhere in this issue (see Professional Status of Audiology in U.S. Healthcare).

As a result of these compounding classification constraints, the coverage of audiology services within the Medicare system is structurally limited in ways that do not apply to comparable healthcare professions. Beneficiaries seeking audiology services under Medicare Part B encounter access barriers not experienced by beneficiaries seeking equivalent services through other provider types, including restricted access to telehealth delivery, which has expanded substantially for other professions in recent years but remains constrained for audiologic care due to the diagnostic-only classification. Addressing these classification constraints at the federal level is the focus of the legislative reform analysis that follows.

### Medicare's Definition of Qualified Audiologist Includes Individuals with Master's or Doctoral Degree

The 1994 amendments defined “qualified audiologist” as the following:

“…an individual with a master's or doctoral degree in audiology who—(i) is licensed as an audiologist by the State in which the individual furnishes such services, or (ii) in the case of an individual who furnishes services in a State which does not license audiologists, has successfully completed 350 clock hours of supervised clinical practicum (or is in the process of accumulating such supervised clinical experience), performed not less than 9 months of supervised full-time audiology services after obtaining a master's or doctoral degree in audiology or a related field, and successfully completed a national examination in audiology approved by the Secretary.” 31

Notably, this definition has never been updated to require doctoral-level preparation despite the profession's formal transition to the AuD as the entry-to-practice degree beginning in 2007. Medicare continues to recognize master's-level audiologists as fully qualified under the program—a reflection of the same policy stagnation that has left audiology's broader classification structure unchanged across three decades of educational and clinical advancement.

### Medicare Audiologist Reimbursement: 100% of the Medicare Physician Fee Schedule; Incident-to Billing Prohibited

A 2008 CMS policy update was communicated through a transmittal prohibited coverage of audiological diagnostic tests under the incident-to physician benefit, affirming that physician supervision is not required for coverage of audiology services delivered by an audiologist and reinforcing the expectation that audiologists practice autonomously within their recognized service category. 34 Audiologists are reimbursed at 100% of the Medicare Physician Fee Schedule (MPFS), a rate consistent with other clinical doctoring professionals including medical doctors, doctors of osteopathy, psychologists, dentists, optometrists, podiatrists, and chiropractors. However, this rate parity applies only to the diagnostic service codes audiologists are authorized to bill. It does not confer access to the evaluation and management codes, rehabilitative billing categories, or novel payment models available to the physician, practitioner, and therapist categories under which other professions are classified. The practical consequence is that reimbursement rate equivalence coexists with fundamental reimbursement scope inequality—audiologists receive the same percentage of the fee schedule as physicians for the services they are permitted to bill, while remaining structurally excluded from the billing mechanisms through which the majority of clinical value is captured in a comprehensive patient encounter.

The incident-to prohibition carries a secondary implication worth noting. While it appropriately prevents the requirement of physician supervision for audiology services, it also means audiologists cannot utilize the incident-to billing arrangements that other non-physician providers use to generate billable revenue under a supervising physician's National Provider Identifier (NPI) in physician-owned practices. This is a constraint as well as a protection, and its net effect on audiology's economic positioning within integrated practice settings warrants acknowledgment.

### Audiologists Categorized as Medicare Suppliers: A Medicare Outlier

To understand the specific nature of audiology's classification disadvantage, it is useful to examine how other healthcare professions are categorized within Medicare's provider framework. Table 2 presents the Medicare category and governing Social Security Act section for 16 provider types, spanning doctoral and sub-doctoral professions, clinical and therapeutic roles, and physician and non-physician practitioners. The table reveals a clear and consistent pattern: every other independently practicing provider—including those without doctoral training, and including master's-level professions such as clinical social workers, nurse practitioners, physician assistants, and SLPs—holds physician, practitioner, or therapist status. Audiology alone is classified as a supplier. 35

**Table 2:** Medicare provider type, degree requirements, and Medicare category under the Social Security Act

Provider type	Clinical doctorate required for practice?	Medicare category	Referenced section of the Social Security Act
Audiologist	Yes	Supplier	1848(k)(3)(B)
Chiropractor	Yes	Physician	1861(r)
Clinical social worker	No	Practitioner	1842(b)(18)(c)
Dentist	Yes	Physician	1861(r)
Dietician	No	Practitioner	1842(b)(18)(c)
Doctor of osteopathy	Yes	Physician	1861(r)
Marriage and family therapist	No	Practitioner	1842(b)(18)(c)
Medical doctor	Yes	Physician	1861(r)
Occupational therapist	No	Therapist	1861(s)
Optometrist	Yes	Physician	1861(r)
Nurse practitioner	No	Practitioner	1842(b)(18)(c)
Physical therapist	Yes	Therapist	1861(s)
Physician assistant	No	Practitioner	1842(b)(18)(c)
Podiatrist	Yes	Physician	1861(r)
Psychologist	Yes	Practitioner	1842(b)(18)(c)
Speech-language pathologist	No	Therapist	1861(s)

As Table 2 illustrates, audiology is the only doctoral-level clinical profession in the Medicare system that does not hold physician, practitioner, or therapist status—a classification that places audiologists in a separate administrative category from every other independently practicing doctoral health provider and from master's-level professions that carry full practitioner or therapist recognition. Audiologists are not included on the list of Medicare-recognized physicians under Section 1861(r), therapists defined under Section 1861(s), or the list of Medicare-recognized practitioners under Section 1842(b)(18)(c) of the Social Security Act.

Instead, audiologists are classified as suppliers under Section 1848(k)(3)(B), listed separately as a category of eligible professional as follows:

“(3) Covered professional services and eligible professionals defined. —For purposes of this subsection:

(A) Covered professional services. —The term ‘covered professional services’ means services for which payment is made under, or is based on, the fee schedule established under this section and which are furnished by an eligible professional.
(B) Eligible professional. —The term ‘eligible professional’ means any of the following:
  (i) A physician.
  (ii) A practitioner described in section 1842(b)(18)(C).
  (iii) A physical or occupational therapist or a qualified speech-language pathologist.
  (iv) Beginning with 2009, a qualified audiologist (as defined in section 861(ll)(3)(B)).”

The practical consequences of this classification extend beyond administrative categorization. When Medicare issues policy updates affecting physicians, practitioners, and therapists—including coverage expansions, billing guidance revisions, telehealth policy changes, and value-based payment model inclusions—audiologists are routinely excluded by default, requiring separate and subsequent policy actions to achieve equivalent recognition. This structural exclusion imposes ongoing administrative burden, introduces coverage gaps that harm beneficiaries, and signals to payers, health systems, and policymakers that audiology occupies a distinct and lesser tier within the federal healthcare system. The economic consequences of this supplier classification—including exclusion from E/M billing, restricted access to rehabilitative reimbursement categories, and structural dependence on referral-based care models—are examined through a labor economic lens in this issue (see Audiology at the Intersection of Labor, Revenue, and Professional Taxonomy).

### Summary

Medicare's classification of audiology services reflects policy decisions made before the profession's transition to doctoral-level entry and has not been updated to reflect the expanded training, clinical scope, or public health role that the AuD degree represents. Audiologists remain the only doctoral-level independent healthcare providers in the Medicare system classified as suppliers rather than physicians, practitioners, or therapists—a designation that restricts access to evaluation and management billing, excludes rehabilitative and chronic-care services from coverage, and prevents participation in the value-based payment models that increasingly govern reimbursement across healthcare. The definition of “qualified audiologist” under Medicare continues to recognize master's-level preparation as sufficient for program participation, reflecting the same policy inertia that has left audiology's classification unchanged despite nearly three decades of educational and clinical advancement. The evidence assembled here is consistent with these classification conditions functioning as structural prerequisites for audiology's professional sustainability and autonomy—conditions whose correction is necessary, though not independently sufficient, for the profession to meet the population health demands documented throughout this issue.

### Traditional Medicare Beneficiaries Have Limited Direct Access to Audiology Services

From 1965 through 2022, traditional Medicare beneficiaries with a suspected hearing or balance problem were required to obtain a physician diagnostic order prior to seeking care from an audiologist—a requirement consistent with Medicare's classification of audiology services as diagnostic tests, a category that includes laboratory tests and imaging, both of which similarly require an order. Beginning January 2023, CMS implemented a partial exception to the physician order requirement, allowing beneficiaries direct access to an audiologist for diagnostic services related to non-acute and non-vestibular hearing conditions and implanted auditory prosthetic devices, once every 12 months on a rolling cycle. 36 A physician order remains required for beneficiaries seeking care for disequilibrium and acute hearing loss under Section 42 CFR 410.32(a)(4). 36

The 2023 policy change, while intended to streamline access to audiology services, has not achieved that objective in practice. The conditional structure of the exception—direct access permitted for some conditions but not others, subject to a 12-month rolling cycle, with order requirements varying by diagnosis—proved too complex for both beneficiaries and providers to navigate consistently, resulting in most beneficiaries continuing to obtain a physician order prior to visiting an audiologist regardless of the clinical indication. A partial reform that generates confusion is not a functional solution. The persistence of access barriers under the 2023 policy underscores the case for the unconditional direct access that practitioner reclassification under the Medicare Audiology Access Improvement Act would provide.

The practical consequences of audiology's current Medicare classification—relative to other provider types—are summarized in Table 3 . The table presents, for 13 provider categories, the Medicare classification, opt-out eligibility, order and referral requirements, and reimbursement rate as a percentage of the MPFS.

**Table 3:** Recognized Medicare provider taxonomy for select Medicare providers

Profession	Classification in Medicare	Can opt out of Medicare?	Order/Referral required for Medicare coverage?	Paid at 100% of the MPFS
Audiologist	Supplier	No	It depends on the reason for the visit *	Yes
Chiropractor	Physician	No	No	Yes
Clinical social worker	Practitioner	Yes	No	No (75%) 45
Dentist	Physician	Yes	No	Yes
Advanced practice nurse	Practitioner	Yes	No	(85%) 46
Occupational therapist	Therapist	No	Order/referral is not required for evaluation or creation of care plan for outpatient services unless required by state law; care plan must be certified by a physician within 30 days. 47	Yes
Optometrist	Physician 48	Yes	No	Yes
Orthotists and prosthetists	Supplier	No	Yes	N/A (Durable medical equipment reimbursed differently) 49
Physician assistant	Practitioner 50	Yes	No	No (85%) 51
Physical therapist	Therapist	No	Order/referral is not required for evaluation or creation of care plan for outpatient services unless required by state law; care plan must be certified by a physician within 30 days. 47	Yes
Podiatrist	Physician	Yes	No	Yes
Psychologist	Practitioner	Yes	No	Yes
Speech-language pathologist	Therapist	No	Order/referral is not required for evaluation or creation of care plan for outpatient services unless required by state law; care plan must be certified by a physician within 30 days. 47	Yes

Abbreviation: MPFS, Medicare Physician Fee Schedule.

* Required for beneficiaries with suspected acute hearing problems, suspected vestibular problems, and those who have been to the audiologist without an order for other issues within a rolling 12–month period.

a An order is not required for non-acute hearing problems if the beneficiary has not seen an audiologist in the last 12 rolling months.

Table 3 reveals a pattern of systematic classification disadvantage. Audiologists cannot opt out of Medicare—meaning they have no mechanism to bill patients directly at market rates outside the program's fee schedule constraints—while chiropractors, dentists, optometrists, podiatrists, and psychologists, all holding physician or practitioner status, retain this flexibility. Audiologists face conditional order requirements that no other provider in the table faces in the same form. And while audiologists are reimbursed at 100% of the MPFS for the diagnostic codes they are authorized to bill, that rate equivalence coexists with categorical exclusion from the billing pathways through which other providers at the same percentage capture the full scope of a clinical encounter. The inability to opt out of Medicare is particularly consequential for private practice sustainability: where other doctoral-level providers can establish direct-pay relationships with patients for services Medicare does not cover, audiologists practicing within the Medicare system have no equivalent pathway.

### The Medicare Audiology Access Improvement Act Offers a Legislative Policy Solution to Improve Access to Medicare Audiology Services

The classification constraints documented throughout this article—diagnostic-only coverage, supplier designation, conditional direct access, exclusion from rehabilitative billing categories, and inability to opt out of Medicare—are not individually intractable. Each maps directly onto a provision of the Medicare Audiology Access Improvement Act (MAAIA), which offers a coordinated legislative remedy for the structural misalignments this article has documented.

The Medicare Audiology Access Improvement Act of 2025, introduced by Senator Warren (D-MA), Chuck Grassley (R-IA), and Senator Rand Paul (R-KY), will update Medicare statutes to expand coverage policies and access through the following provisions:

- Amends the definition of “audiology services” in the Medicare statute to include all services already covered by Medicare that are also within an audiologist's scope of practice (cerumen removal, auditory rehabilitation, and vestibular rehabilitation);
- Reclassifies audiologists from supplier to practitioner under the Medicare statute, allowing them to be more readily deployed;
- Allows beneficiaries to have coverage of audiology services when accessed via telehealth. The practitioner classification is consistent with the classification of similar healthcare providers such as clinical social workers, nurse practitioners, physician assistants, and clinical psychologists;
- Removes the diagnostic order requirement for all covered audiology services, allowing beneficiary direct access to audiologists;

- Ensures seniors and people with disabilities can receive the full scope of audiology services covered by Medicare at Rural Health Centers and Federally Qualified Health Centers; and
- Makes no changes to the scope of benefits covered by Medicare or the scope of practice for audiologists. Audiologists' scope of practice is determined entirely by state law.

The legislation, shepherded by the Academy of Doctors of Audiology, the American Academy of Audiology, and the American Speech-Language-Hearing Association, has achieved bipartisan support in Congress and is broadly supported by consumer and multidisciplinary provider organizations including American Association for Retired Persons (AARP), the American Tinnitus Association, the National Association of Rural Health Clinics, the National Rural Health Association, and the Vestibular Disorders Association.

The clinical model that MAAIA would formalize within Medicare has already been validated in integrated healthcare systems. Within the Veterans Health Administration—the largest integrated health system in the United States—audiologists function with expanded clinical authority, independent entry points, and direct patient access across the full scope of auditory and vestibular care, demonstrating improved patient outcomes and reduced utilization-related costs. 52 Expert respondents in the qualitative analysis presented in Audiology in 2050 of this issue consistently identified the VA model not as an aspirational ideal but as an operational reality already functioning at scale—evidence that the policy barriers to full-scope audiology practice are the only remaining obstacle separating current Medicare policy from a model that demonstrably works. 52 Aligning Medicare policy with this proven model, through MAAIA's reclassification and coverage expansion provisions, would improve pathways to care for the Medicare beneficiaries who bear the greatest burden of untreated hearing and balance disorders.

Importantly, MAAIA's effectiveness as a policy instrument depends on concurrent progress in scope modernization and standardized practice. Reclassification that arrives ahead of consistent state scope alignment will be uneven in implementation; federal coverage expansion that precedes workforce models capable of capturing its value will leave structural gains partially unrealized. As the concluding analysis in the article Professional Status of Audiology in U.S. Healthcare: A Health Economics Perspective of this issue demonstrates, MAAIA functions most powerfully as a multiplier of upstream alignment rather than as a standalone solution—amplifying the professional authority that scope modernization, educational reform, and standardized practice make credible, and translating it into the reimbursement recognition the profession's training and public health role warrant.

### Summary

Medicare's classification of audiology services has remained substantively unchanged since the program's inception, despite the profession's transition to doctoral-level practice, the expansion of audiology's clinical scope, and the accumulation of evidence linking hearing and balance care to broader health outcomes across the lifespan. The treatment vacuum, supplier designation, conditional direct access, and exclusion from opt-out eligibility and value-based payment models documented in this section are not isolated policy anomalies—they are the coherent expression of a classification structure that has never been updated to reflect what audiology is, what audiologists are trained to do, or what Medicare beneficiaries need from the profession. MAAIA represents the most comprehensive legislative effort to correct these structural misalignments and to position audiology within Medicare as the autonomous, doctoral-level clinical profession it has become. Its passage is a necessary—though not independently sufficient—condition for audiology to achieve the professional standing and workforce sustainability that the analyses presented throughout this special issue identify as both attainable and urgent.

## Discussion and Conclusion

The evidence assembled in this article points toward a conclusion that is both straightforward and consequential: the barriers to audiology's full professional recognition are not primarily clinical, educational, or market-driven, they are statutory and regulatory. State practice acts define audiology in language that predates the AuD, qualifies its activities as nonmedical, restricts the use of assistants, and omits the terms—diagnose, manage, treat, prescribe—that insurance systems use to determine coverage eligibility and reimbursement authority. Medicare classifies audiology services as diagnostic-only and audiologists as suppliers, designations that have not been updated since 1994 despite the profession's transition to doctoral-level entry, the expansion of its clinical scope, and the accumulation of evidence linking hearing and balance care to outcomes across the full spectrum of adult and pediatric health. These constraints do not operate independently. They form a mutually reinforcing system: outdated state scope language provides the regulatory justification for narrow federal classification; narrow federal classification suppresses the economic authority that would otherwise incentivize state scope modernization; and the resulting treatment vacuum, E/M exclusion, and reimbursement limitations compound into workforce consequences—constrained earnings, geographic maldistribution, and accelerating substitution by less-trained providers—that the companion analyses in this issue document in detail.

The consequences of this classification system extend beyond the Medicare program. Because private payers routinely adopt Medicare coverage determinations as the baseline for their own benefit design, audiology's diagnostic-only classification under Medicare effectively sets the ceiling for coverage across both public and private insurance markets. The treatment vacuum documented in this article—the exclusion of vestibular rehabilitation, aural rehabilitation, and cerumen management from reimbursed audiology services—is not a Medicare-specific anomaly. It is a systemic limitation that cascades through private insurance benefit structures, affecting access to audiologic care for patients across all payer categories. The magnitude of this downstream effect has not been fully quantified in the audiology literature and represents an important direction for future health services research.

State audiology practice acts and Medicare policies also interact in a second important way: the absence of modernized scope language at the state level provides cover for the perpetuation of restrictive federal classification. When state statutes define audiology as the nonmedical application of principles and procedures, and when fewer than half of states include the terms diagnose, manage, or treat in their definitions of audiology practice, federal policymakers and private payers have statutory grounds for treating audiology services as technical rather than clinical in nature. Removing the “nonmedical” qualifier, incorporating the full diagnostic and management vocabulary into state practice acts, and aligning scope language with AuD accreditation standards—as the article Shaping the Future of Audiology: Current and Evolving Landscape of Audiology Education of this issue examines directly—are therefore not merely symbolic reforms. They are the regulatory preconditions for federal reclassification and expanded reimbursement authority to function as intended.

The profession's capacity to pursue this agenda is itself a structural variable. In the past, audiology advocacy has been fragmented across multiple associations representing different segments of the profession, with divided resources, inconsistent messaging, and competitive rather than complementary legislative strategies. The evidence assembled in this issue is consistent with fragmentation functioning as a structural liability—one that has allowed classification constraints to persist for decades despite their well-documented consequences for patients, practitioners, and the profession's long-term viability. The dependency analysis developed in Audiology in 20250: Aligning Collective Professional Perspectives to Create a Roadmap for the Future of this issue predicts that coordinated effort across the profession's representative organizations—with unified messaging, shared resources, and sequencing that prioritizes scope modernization and standardized practice as enabling conditions for federal reclassification—is more likely to produce durable reform than fragmented or parallel legislative strategies.

The statutory and regulatory gaps documented in this analysis point toward two specific policy priorities whose resolution the evidence supports as necessary conditions for addressing disadvantages of audiology's classification:

- State audiology practice acts are most closely aligned with the full competency expectations of doctoral-level training when they incorporate the terms “evaluate, diagnose, manage, prescribe [hearing aids], and treat auditory or vestibular conditions in the human ear”; remove “nonmedical” qualifiers from the definition of audiology services; eliminate prohibitions on the use and supervision of audiology assistants for clinical services including hearing aid dispensing and fitting; and include additional amendments commensurate with audiologists' doctoral-level education and training. The Maryland and Arkansas statutes represent the current legislative benchmark for this alignment.
- MAAIA addresses each of the classification constraints documented in this article—supplier designation, the treatment vacuum, conditional direct access, telehealth exclusion, and rural health coverage gaps—through a coordinated set of statutory provisions. The evidence presented here identifies these constraints as structural rather than incidental, which is consistent with their resolution requiring legislative action at the federal level rather than administrative adjustment alone.

Public policies defining the practice of audiology must be modernized as a coordinated system rather than as a series of isolated reforms. The nomenclature and taxonomy barriers documented in this article are examined in greater depth across companion articles in this special issue, including Audiology at the Intersection of Labor, Revenue, and Professional Taxonomy and Professional Status of Audiology in U.S. Healthcare: A Health Economics Perspective. In addition, the article Audiology in 2050: Aligning Collective Professional Perspectives to Create a Roadmap for the Future synthesizes collective professional perspectives to map the coordinated reform sequence–scope modernization, educational alignment, and federal reclassification–through which the profession's classification, authority, and utilization can be brought into structural alignment with its training and public health responsibilities.
